# Betacoronavirus internal protein: role in immune evasion and viral pathogenesis

**DOI:** 10.1128/jvi.01353-24

**Published:** 2025-01-06

**Authors:** Chaminda D. Gunawardene, Lok-Yin Roy Wong

**Affiliations:** 1 Center for Virus-Host Innate Immunity, Rutgers New Jersey Medical School 5970 , Newark, New Jersey, USA; 2 Department of Microbiology, Biochemistry and Molecular Genetics, Rutgers New Jersey Medical School 5970 , Newark, New Jersey, USA; Universiteit Gent, Merelbeke, Belgium

**Keywords:** virology, coronavirus, pathogenesis, immune evasion, internal protein

## Abstract

Betacoronaviruses express a small internal (I) protein that is encoded by the same subgenomic RNA (sgRNA) as the nucleocapsid (N) protein. Translation of the +1 reading frame of the N sgRNA through leaky ribosomal scanning leads to expression of the I protein. The I protein is an accessory protein reported to evade host innate immune responses during coronavirus infection. Previous studies have shown that the I proteins of severe acute respiratory syndrome coronavirus (SARS-CoV), SARS-CoV-2, and Middle East respiratory syndrome coronavirus suppress type I interferon production by distinct mechanisms. In this review, we summarize the current knowledge on the I proteins of betacoronaviruses from different subgenera, with emphasis on its function and role in pathogenesis.

## INTRODUCTION

**Fig 1:**
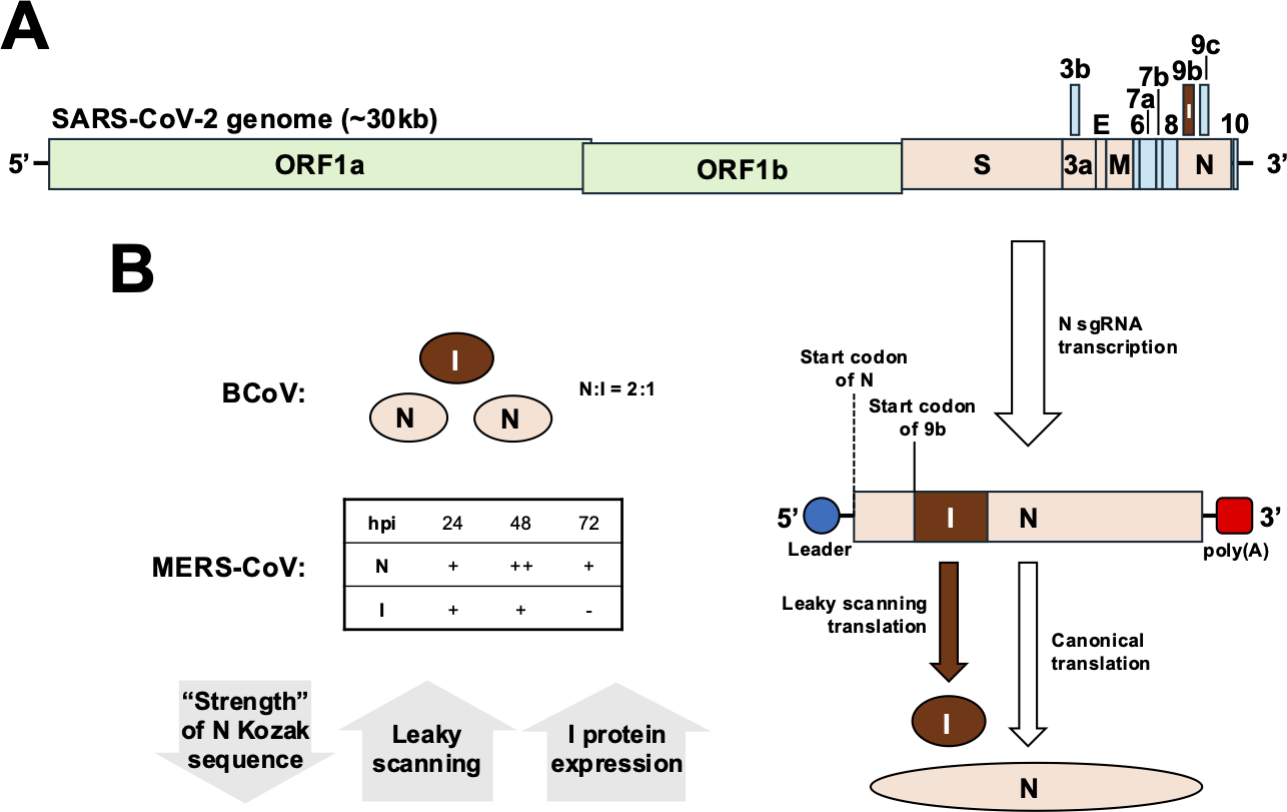
Expression strategy of betacoronavirus internal protein. (**A**) Schematic diagram depicting the genome organization of severe acute respiratory syndrome coronavirus 2 (SARS-CoV-2). SARS-CoV-2 is used as an example to illustrate the expression of the I protein (9b). (**B**) Schematic diagram showing the expression of the N and I proteins. Subgenomic RNA (sgRNA) expressing the N protein is depicted. Leaky ribosomal scanning happens when translation is not initiated at the start of codon of the N protein. Instead, translation is initiated at the start codon of 9b, leading to the expression of the protein. The relative abundance of the internal protein expression relative to the N protein is plausibly determined by the “strength” of the Kozak sequence for the initiation of N protein translation. The *Bovine enteric coronavirus* (BCoV) I protein:N protein expression has been previously determined as 1:2 at molar ratio (8). The levels of the Middle East respiratory syndrome coronavirus (MERS-CoV) I protein expression were detected in infected cells (1, 9). The relative abundance of N and I protein expression in infected cells is illustrated as a table. “+” indicates that expression of the protein was detected, “++” indicates peak expression, and “−“ indicates extremely low levels or not detectable expression.

Coronaviruses (CoVs) are positive, single-stranded, enveloped RNA viruses that encode one of the largest viral RNA genomes (~30 kb) known to date. CoVs express three types of viral proteins upon infection, which are broadly categorized as structural proteins, non-structural proteins (nsps), and accessory proteins (Fig. 1). While structural proteins and nsps are indispensable for the viral life cycle, accessory proteins are not essential for virus replication and productive infection. In fact, accessory proteins often act as virulence factors to enhance virus replication and pathogenesis, mainly by virtue of their immunoevasive properties (1–6). Accessory proteins are minimally conserved among CoVs of different subgenera compared to nsps and structural proteins, which may partially explain the disparity in the pathogenicity of different CoVs. However, CoVs within the same subgenus (also previously known as lineage/group) often encode a set of unique and similar accessory proteins (7). Hence, accessory proteins are also referred to as lineage-specific proteins. In particular, CoVs in different genera of the *Betacoronavirus* genus express a small accessory protein, the internal (I) protein, that suppresses host innate immune responses during infection. Although I protein sequences are more conserved within each subgenus but not across subgenera, betacoronaviruses of different subgenera often encode the I gene at a similar genomic location that overlaps with the N gene. Structural modeling and analysis reveal that some I proteins of different subgenera share similarities in their putative structures, even with low degree of protein sequence similarity, suggesting that the I protein could be an accessory protein with conserved functions. Here, we summarize the current knowledge on the molecular functions of the betacoronavirus I protein in relation to pathogenesis and discuss future research directions.

## OVERVIEW OF BETACORONAVIRUS INTERNAL PROTEIN

The I protein was first described in *Bovine enteric coronavirus* (BCoV) of the subgenus *Embecovirus,* which is known to infect cattle and other ruminants (10–12). Initial sequence analyses posited that the I protein is expressed by a putative open reading frame (ORF) overlapping with the nucleocapsid (N) gene (11). It was later confirmed that the BCoV N subgenomic RNA (sgRNA) is bicistronic, and that translation of the BCoV N sgRNA during BCoV infection leads to the expression of the N and I proteins (12). Subsequent studies with mouse hepatitis virus (MHV) showed that the I protein is a structural protein detected in the virions. However, recombinant viruses lacking I protein expression did not result in diminished virus replication or ameliorated disease in mice (13). Furthermore, human betacoronaviruses from different subgenera including human coronavirus HKU1 (HCoV-HKU1), HCoV-OC43 (*Embecovirus*), severe acute respiratory syndrome coronavirus (SARS-CoV), SARS-CoV-2 (*Sarbecovirus*), and Middle East respiratory syndrome coronavirus (MERS-CoV) (*Merbecovirus*) encode this internal gene within the N sgRNA (7, 14–19). The betacoronavirus I protein is primarily recognized as an interferon (IFN) antagonist. The molecular mechanisms of IFN antagonism and roles in pathogenesis have been reported for certain I proteins (1–3, 20). The current nomenclature for this protein is not standardized between CoVs, resulting in a variety of different names being used for the protein across the *Betacoronavirus* genus (e.g., N2 for HCoV-HKU1 and HCoV-OC43, 9b for SARS-CoV and SARS-CoV-2, 8b for MERS-CoV, I for MHV). For simplicity, we will refer to this gene/protein (the first downstream ORF in the +1 open reading frame relative to the N gene and its translation product) as the I gene/protein in this review.

## TRANSLATION STRATEGY FOR I PROTEIN EXPRESSION

The strategy of encoding multiple viral proteins within a single gene is not unique to coronaviruses. Other RNA viruses also express multiple gene products translated by alternative reading frames embedded within an overlapping, polycistronic sgRNA. For example, the influenza virus PB1 gene encodes another gene that leads to the expression of a second, smaller protein PB1-F2 in addition to the RNA-dependent RNA polymerase PB1 (21). The measles virus P gene encodes for three gene products, the phosphoprotein (P), C, and V proteins (22, 23). Both influenza virus PB1-F2 and measles virus C proteins are expressed by translating the +1 reading frame of the PB1 and P genes, respectively (21, 23). Similarly, the expression of betacoronavirus I protein is dependent on the N sgRNA, as no canonical I-specific transcription regulatory sequence (TRS) has been identified. The I gene shares the same TRS and is translated from the +1 reading frame relative to the N gene (14, 17, 24). Although the exact mechanism that leads to I protein expression remains elusive, indirect evidence suggests that the I protein is expressed by leaky ribosomal scanning (Fig. 1). Sequence comparison revealed that a mutation identified in SARS-CoV-2 variants that weakens the Kozak sequence for N sgRNA translation was sufficient to increase the levels of I protein expression in infected cells (9, 24). Moreover, a substitution from aspartic acid to leucine at residue 3 of the N protein (D3L) generated a non-canonical TRS-like element upstream of the coding sequence of the SARS-CoV-2 I protein, leading to the transcription of an I sgRNA independent of the N sgRNA (25). Although the D3L mutation in the N protein led to increased I-specific sgRNA expression, this did not result in elevated SARS-CoV-2 I protein levels in infected cells, further corroborating the notion that the I protein is primarily expressed through leaky ribosomal scanning of the N sgRNA. Furthermore, the abundance of the I protein varies during infection for different betacoronaviruses. Previous studies using *in vitro* translation assays demonstrated that the I protein was produced at approximately half the molar ratio relative to the N protein for BCoV (8). A previous study showed that the expression levels of MERS-CoV I protein remain relatively constant with increasing levels of N protein in infected cells early during infection but wane at a later stage of infection (9). MHV-infected cells also express the I protein early after infection with diminishing levels of the I protein late in infection (5), suggesting that the I protein may have a functional role at early stages of infection. Immunohistochemical studies showed that SARS-CoV I protein was present at detectable levels in infected cells and clinical samples obtained from SARS-CoV-infected patients (26). However, I protein expression was only detected in cells infected with SARS-CoV-2 encoding the mutation in the N protein Kozak sequence that enhances I protein translation by leaky ribosomal scanning but not in wild-type (WT) SARS-CoV-2-infected cells, suggesting that the SARS-CoV-2 I protein is not abundantly produced during infection (9). Consistently, other studies showed that the I protein was expressed at lower levels in cells infected with ancestral strains of SARS-CoV-2 compared to that infected with SARS-CoV-2 variants encoding the mutation that weakens the N protein Kozak sequence (25). These data suggest that the I protein is expressed at varying levels during infection for different betacoronaviruses. Future studies are warranted to understand the dynamics and mechanisms of I protein expression during infection in relation to their role in pathogenesis.

## I PROTEIN FUNCTION AND MOLECULAR INTERACTIONS

### IFN-I antagonism

**Fig 2:**
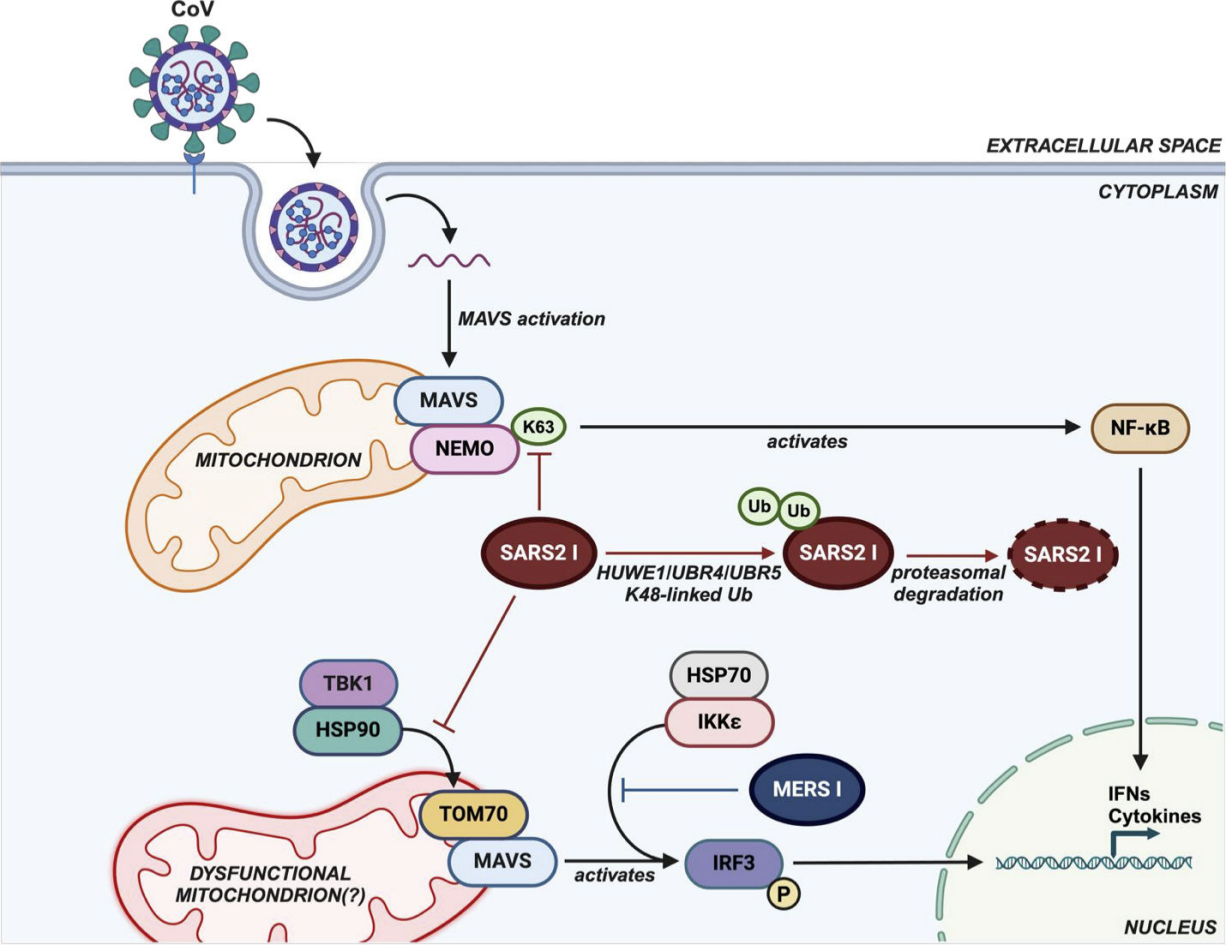
Functions of SARS-COV-2 I protein and its interactions with the host. Coronaviruses interact with cellular receptors for entry. Upon uncoating, the viral genomic materials activate the host RNA-sensing pathway, resulting in the activation of MAVS. Downstream signaling of MAVS requires (i) the activation of nuclear factor κB (NF-κB) essential modulator (NEMO) for inducing NF-κB-dependent gene expression and (ii) the recruitment of TBK1 for IRF3-dependent transcriptional activation. The SARS-CoV-2 I protein (SARS2 I) has been reported to inhibit K63-linked ubiquitination of NEMO, leading to the suppression of NF-κB activation (3). SARS2 I also allosterically disrupts the interaction between HSP90 and TOM70, which is critical for the recruitment of TBK1 to the MAVS signaling complex for IRF3 activation (2, 45). The presence of SARS2 I leads to the downregulation of IRF3-dependent and NF-κB-dependent gene expressions including IFNs and cytokines. The interaction between SARS2 I with TOM70 may lead to mitochondrial dysfunction as TOM70 is known to mediate protein transport across the mitochondrial membrane (49, 50). In addition, host E3 ubiquitin ligases HECT, UBA, and WWE domain-containing E3 ubiquitin protein ligase 1 (HUWE1), ubiquitin protein ligase E3 component n-recognin 4 (UBR4), and UBR5 induce K48-linked ubiquitination of SARS2 I and lead to its degradation (47). The MERS-CoV I protein (MERS I) was reported to interact with HSP70. This prevents HSP70-dependent IKKε activation and hence suppression of IRF3 phosphorylation and IFN induction (1). This figure was created with BioRender.

Coronaviruses are known to evade host innate immune responses upon infection. Suppressing host IFN induction and signaling is a common strategy employed by coronaviruses to establish infection. Delayed and dysregulated IFN induction and signaling are commonly observed in severe disease for experimentally infected animals and during human infections (2, 27–34). Multiple viral proteins encoded by coronaviruses have been reported to block IFN induction and signaling (1, 2, 35–41). Among betacoronaviruses, the I protein is characterized as an IFN-I antagonist. Multiple studies reported that the I protein of different betacoronaviruses inhibits IFN-I expression through distinct mechanisms (1–3, 20, 42, 43). Ectopic expression of the SARS-CoV I protein is reported to suppress IFN-I induction by inducing ubiquitin-mediated proteasomal degradation of the mitochondrial antiviral signaling (MAVS) protein complex, which is important for transcriptional activation of IFN-I (20). Overexpression of the SARS-CoV-2 I protein impedes IFN-I expression by interacting with translocase of outer membrane 70 (TOM70) (2) (Fig. 2). TOM70 is a mitochondrial import receptor required for MAVS-mediated IFN-I induction. TOM70 associates with MAVS upon RNA virus infection and recruits TANK-binding kinase 1 (TBK1) to form the MAVS/TBK1 signaling complex for interferon regulatory factor 3 (IRF3) activation and IFN-I induction. The recruitment of TBK1 by TOM70 is mediated through their interactions with heat shock protein (HSP) 90 (44, 45). Binding of SARS-CoV-2 I protein to TOM70 impairs the interaction between TOM70 and HSP90, preventing the formation of the MAVS/TBK1 complex and subsequent IFN-I induction (2, 46). Importantly, two independent studies have identified a key residue responsible for the I protein interaction with TOM70. Phosphorylation of the serine residue at position 53 (Ser53) or phosphomimetic substitutions in the SARS-CoV-2 I protein significantly reduced I protein binding to TOM70, resulting in the loss of SARS-CoV-2 I protein-mediated suppression of IFN-I responses (24, 46). A recent study reported that SARS-CoV-2 I protein is degraded through K48-linked ubiquitination, which is induced by specific host E3 ubiquitin ligases, potentially as an antiviral measure counteracting the function of the protein (47). This also suggests that the I protein may alter the host ubiquitination pattern by sequestering host ubiquitin ligases from their cognate targets. Furthermore, SARS-CoV-2 I protein has been reported to antagonize IFN induction mediated by retinoic acid-inducible gene I-like receptor, toll-like receptor, and the cyclic GMP–AMP synthase–stimulator of interferon genes pathways (3, 48). These results indicate that SARS-CoV-2 I protein is an IFN antagonist that targets multiple host pathways for immune evasion.

Similar to the SARS-CoV and SARS-CoV-2 I proteins, the MERS-CoV I protein functions as an IFN antagonist. The MERS-CoV I protein has been shown to inhibit the IFNβ promoter-driven gene expression induced by the synthetic double-stranded RNA (dsRNA) analog polyinosinic-polycytidylic acid (poly I:C) and virus infection upon ectopic expression (1, 40, 51). Mechanistically, MERS-CoV I protein competitively sequesters inhibitor of nuclear factor kappa-B kinase subunit epsilon (IKKε), preventing binding between IKKε and HSP70. Since IKKε interaction with HSP70 enhances its activation by autotransphosphorylation, the presence of MERS-CoV I protein leads to dampened IKKε activation and hence diminished IFNβ expression (1). In addition, deletion of the N-terminus (residues 1–23) of the MERS-CoV I protein did not result in loss of IFN suppression, suggesting that the C-terminal region of the I protein is critical for IFN antagonism (52). The interaction of the MERS-CoV I protein with HSP70 prompted the possibility that TOM70 may be involved in MERS-CoV I protein-mediated IFN antagonism, as HSP70 is known to associate with TOM70 as part of a translocation complex for importing mitochondrial protein (49). A previous study also suggested that TOM70 interacts with the MERS-CoV I protein upon ectopic expression (9) but to a lesser extent compared to the SARS-CoV-2 I protein. Future investigations on the functions of betacoronavirus I protein with respect to TOM70 and HSPs will provide valuable insights on the conserved strategies exploited by different betacoronaviruses to evade host immune responses.

### Alternative functions and putative role as a structural protein

Coronavirus infection is known to perturb host mitochondrial function. Previous studies have shown that SARS-CoV-2 infection alters nuclear-encoded mitochondrial gene expression in different organs as a result of a systemic response to SARS-CoV-2 infection. These transcriptomic changes lead to inhibition of oxidative phosphorylation and enhanced glycolysis that contribute to disease progression and pathogenesis (53, 54). Aberrant mitochondrial elongation, likely caused by an increase in mitochondrial transmembrane potential, was reported after SARS-CoV-2 infection (55). Furthermore, SARS-CoV-2 infection activates epidermal growth factor receptor -mediated signaling, contributing to mitochondrial dysfunction (55). Despite these findings, a mechanism for coronavirus-mediated mitochondrial alterations remains elusive. Given the interactions between betacoronavirus I protein with TOM70 and HSPs, which are known to associate with mitochondria, the I protein presents a plausible means to mediate such alterations. Ectopic expression of SARS-CoV-2 I protein in cell culture resulted in depletion of mitochondrial proteins, likely caused by the interaction between the I protein and TOM70 (50, 56). This study demonstrated the role of the I protein in blocking mitochondrial protein import and modulating mitochondrial biogenesis, providing a mechanistic basis for the mitochondrial alterations observed. This finding also highlights a function of the I protein in addition to immune evasion, underscoring the multifunctional nature of the I protein. However, the I proteins of the neurotropic JHM strain of MHV (JHMV) and MHV-A59 do not co-localize with TOM70 (5). Therefore, it remains to be investigated whether the modulation of mitochondrial activity is a conserved feature among I proteins of different betacoronaviruses.

*In vitro* screening for SARS-CoV-2 I protein interacting partners revealed its binding to host factors responsible for protein folding, intracellular vesicle trafficking, and endosomal recycling (57, 58). These results highlight that the I protein closely interacts with host membranes to perturb diverse cellular events. In a previous study, the I protein of MHV-A59 was shown to be incorporated into the virion, hinting at its role as a minor structural protein (13). This study provides an additional perspective to study the function of the I protein in addition to the potential immunological role of the I protein. A recent study by Lowery et al. demonstrated that the I protein of JHMV is essential for virion packaging and assembly. JHMV I protein was reported to localize to the Golgi compartment and the endoplasmic reticulum–Golgi intermediate compartment, the primary sites for coronavirus assembly and budding. Infection with JHMV lacking the I protein resulted in reduced infectious virus titers despite similar levels of viral RNA compared to WT JHMV-infected cells, suggesting a defect in virion packaging. Consistently, electron microscopy revealed that cells infected with JHMV lacking the I protein harbor significantly fewer virions (5). These data indicate that JHMV I protein is present in the virion and plays a role in virion assembly. In addition to JHMV and MHV-A59, the I protein of SARS-CoV is also incorporated into the virion in the presence of the envelope (E) and membrane (M) proteins (59), providing further evidence that the I protein acts as a minor structural protein, probably facilitating virion assembly in conjunction with other structural proteins. These findings postulate a putative structural role for the I protein. Moreover, previous reports have shown that other coronavirus accessory proteins are present in the virion and that some are important for evading host-mediated entry inhibition (4). It is thus possible that I protein within the virion may also play additional roles in evading entry inhibition beyond its role in virion assembly, which merits further study.

## ROLE OF THE I PROTEIN IN PATHOGENESIS

Betacoronavirus I proteins have been shown to perturb various host pathways and facilitate virus packaging and assembly (5, 24, 55), suggesting that the I protein functions as a virulence factor that contributes to coronavirus pathogenesis. The analysis of accessory proteins in relation to coronavirus pathogenesis often involves genetic ablation of the protein of interest with reverse genetics and infection study using animal models. This is inherently challenging, as genetic ablation of the I protein would inevitably result in partial deletion of the N protein. To circumvent this, several studies have generated authentic recombinant coronaviruses lacking I protein expression by introducing premature stop codons or mutating start codons in the I protein reading frame without significantly altering N protein expression (9, 13, 25). This is possible as the I protein is translated at an alternative (+1) reading frame relative to the N protein so that non-synonymous mutations in the I protein reading frame may result in synonymous changes in the N protein reading frame. However, one caveat of this approach is that truncated versions of the I protein could be expressed.

The immune evasive property of the I protein implies that viruses lacking I protein expression would be attenuated. Indeed, the deletion of SARS-CoV-2 I protein resulted in the attenuation of virulence and pathogenicity in mice. Mice infected with SARS-CoV-2 lacking I protein exhibited reduced weight loss and improved survival compared to those infected with WT SARS-CoV-2 (9). Consistently, less virus was detected in the lungs of mice infected with the I protein-deficient virus. Previous studies suggested that SARS-CoV-2 variants evolve to enhance immune evasion and that such enhancement is partially contributed by increased I protein levels (24, 25). However, SARS-CoV-2 variants with mutations that result in enhanced I protein expression did not cause exacerbated disease in mice (9), suggesting that other mutations in SARS-CoV-2 variants may play consequential roles in pathogenesis. Genetic ablation of JHMV I protein resulted in significantly ameliorated disease in infected mice, marked by reduced weight loss and clinical symptoms, and a concomitant increase in survival (5). In contrast, a previous study showed that deletion of the SARS-CoV I protein had minimal effects on virulence and pathogenesis in infected mice (60). Similarly, two independent studies reported that deleting the MHV-A59 I protein had minimal effects on virus replication, weight loss, or survival in infected mice (5, 13). This suggests that despite the high degree of sequence similarity between SARS-CoV and SARS-CoV-2 I proteins (~72%) or MHV-A59 and JHMV I proteins (~78%), the I proteins of SARS-CoV-2 and JHMV play more significant roles in pathogenesis than those of SARS-CoV and MHV-A59, respectively.

The effect of the I protein on MERS-CoV pathogenesis has been reported *in vivo*. Genetic knockout of the I protein in a mouse-adapted strain of MERS-CoV resulted in increased weight loss, reduced survival, and more robust virus replication in the lungs of infected mice (9), suggesting that MERS-CoV I protein limits pathogenesis during MERS-CoV infection in mice. These results were highly unexpected, given the immunoevasive nature of MERS-CoV I protein observed *in vitro* (1, 40) and the precedence that removing I proteins from SARS-CoV, SARS-CoV-2, or MHV either did not result in significant changes in virulence or resulted in decreased virulence. On the contrary, the introduction of the MERS-CoV I protein to an attenuated strain of neurotropic JHMV (J2.2) indicated that the I protein contributes to exacerbated encephalitis and clinical disease in infected mice, suggesting that MERS-CoV I protein contributes to pathogenesis in the context of J2.2 infection (9, 52). Further analysis revealed that the N-terminus of MERS-CoV I protein (amino acids 1–23) is critical for the exacerbated pathogenicity observed in J2.2-infected mice. Future investigations could clarify the mechanisms for the disparate roles of the MERS-CoV I protein in relation to pathogenesis in the context of MERS-CoV and J2.2 infection.

## BETACORONAVIRUS I PROTEIN STRUCTURE IN RELATION TO FUNCTION

Currently, there is limited structural information on betacoronavirus I proteins. The I proteins of SARS-CoV and SARS-CoV-2 are the only I proteins with experimentally determined structures. The I proteins of SARS-CoV and SARS-CoV-2 share a high degree of sequence and structural similarity and form symmetrical, homodimeric structures mainly composed of β-strands with a hydrophobic central cavity embedded within the core (57, 61, 62). This hydrophobic tunnel likely contributes to the membrane-interacting properties of the protein. *In silico* analysis predicts that the SARS-CoV-2 I protein harbors an internal mitochondrial targeting signal (MTS) which is preferentially recognized by the C-terminal tetratricopeptide repeats (TRP) motifs of TOM70 (57, 62, 63). Structural analysis revealed that the I protein and TOM70 interact through a hydrogen bond formed between Ser53 at the central part of SARS-CoV-2 I protein and Glu477 at the C-terminal TRP of TOM70. This provides a structural basis for the diminished binding between the I protein and TOM70 when Ser53 is phosphorylated (57, 62, 63). Interestingly, several studies have shown that complex formation was not feasible by combining purified SARS-CoV-2 I protein and TOM70 (57, 62), suggesting that the I protein does not interact with TOM70 in its dimeric structure. However, the I protein complexes with TOM70 in its protomeric form when the two proteins are co-expressed. The protomeric structure of the I protein adopts an alpha-helical fold in the I protein/TOM70 complex (57, 62), which is structurally distinct from the dimeric structure with predominantly β-strands in its native conformation. These data suggest that the transition of SARS-CoV-2 I protein between its dimeric and protomeric forms may modulate its interaction with other proteins and functions. Studies on the mechanisms that mediate the transition are warranted. Furthermore, the N-terminal TRP motifs of TOM70 are known to interact with the C-terminal EEVD motif located in HSPs (49, 64). However, SARS-CoV-2 I protein interaction with the C-terminal TRP motifs of TOM70 drastically reduces the binding affinity between the N-terminal TRP motifs and the EEVD motif of HSP, indicating a robust I protein-mediated allosteric inhibition of HSP association with TOM70 (57).

Since there are currently no experimentally resolved structures for other betacoronavirus I proteins, several studies have attempted to predict and model the structure of MERS-CoV and MHV I proteins (5, 9). Structural prediction indicated that the protomeric forms of the I proteins of JHMV, MHV-A59, and MERS-CoV share similar structures, with an N-terminal disordered linker region, central alpha-helical domain, and C-terminal tail (5). These structures resemble the protomeric structure of the SARS-CoV-2 I protein, which employs a predominantly alpha-helical central domain (57, 62). *In silico* analysis also predicted an internal MTS for MERS-CoV I protein (9). This suggests that I proteins from different subgenera may adopt similar structures despite not sharing highly conserved sequences. However, experimental verification is required to validate the predicted structures.

## PHYLOGENETIC RELATIONSHIPS AMONG BETACORONAVIRUS I PROTEINS

**Fig 3:**
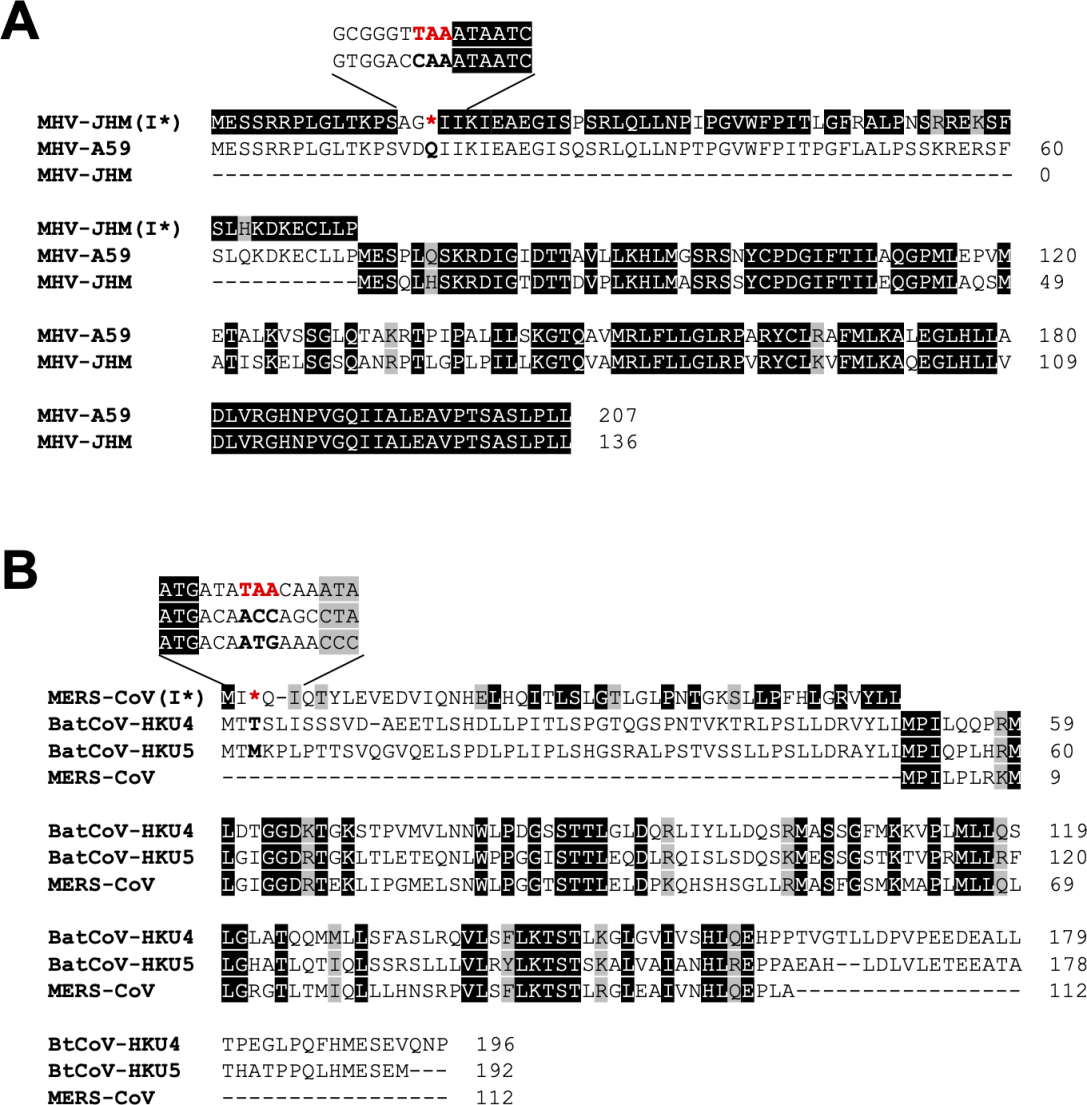
Sequence alignments for I proteins of closely related betacoronaviruses. The I protein sequences of MHV-JHM and MHV-A59 (**A**), MERS-CoV and BatCoV-HKU4/5 (**B**) are aligned and compared. Amino acid sequences upstream of the start codon of the I proteins for MHV-JHM and MERS-CoV are illustrated as MHV-JHM (I*) in panel **A** and MERS-CoV (I*) in panel **B**, respectively. MHV-JHM (I*) and MERS-CoV (I*) are not expressed as part of the MHV-JHM and MERS-CoV I proteins, respectively, due to the presence of a stop codon in the sequence (highlighted in red). These result in the translation of MHV-JHM and MERS-CoV I proteins initiating at a position equivalent to the second start codon of MHV-A59 and BatCoV-HKU4/5 I proteins, respectively. These stop codons are not observed in MHV-A59 (**A**) or BatCoV-HKU4/5 (**B**) I proteins. MHV-JHM (I*) shares a high degree of similarity with the N-terminal part of the MHV-A59 I protein. MERS-CoV (I*) shares moderate similarity with the N-terminal parts of the BatCoV-HKU4/5 I proteins. The C-terminal part of the MERS-CoV I protein is also truncated compared to those of BatCoV-HKU4/5. “–” represents a gap in the sequences.

Sequence similarity of I proteins within the same subgenera is highly similar relative to those of other subgenera, consistent with their classification as accessory proteins. Phylogenetic and sequence analysis revealed possible evolutionary events involving the I protein. I proteins of MHV-A59 and JHMV of the *Embecovirus* subgenera share extensive sequence conservation. However, JHMV I protein is truncated at the N-terminus relative to the MHV-A59 I protein due to the presence of an in-frame stop codon upstream of the start codon of the JHMV I protein (corresponding to the second start codon in the MHV-A59 I protein coding sequence) (5) (Fig. 3). The presence of this stop codon resulted in an N-terminal truncation of 71 residues and lower molecular weight for the I protein of JHMV relative to that of MHV-A59. The molecular weight of the JHMV I protein corresponds to the translation product initiated by the next available methionine residue immediately downstream of the stop codon (5, 13). The N-terminal domain of the I protein of MHV-A59 and the coding amino acid sequence upstream of the start codon of JHMV I protein (corresponding to the second methionine residue in the MHV-A59 I protein) show near identical amino acid composition (Fig. 3A), suggesting that JHMV would yield an almost identical I protein to that of MHV-A59 in the absence of this stop codon (5). This phenomenon is also observed in the *Merbecovirus* subgenus. Bat coronavirus HKU4 (BatCoV-HKU4), HKU5 (BatCoV-HKU5), and MERS-CoV are closely related viruses that each encode I genes within their respective N genes. Sequence alignment of these I proteins showed a moderate degree of conservation. Notably, the I proteins of BatCoV-HKU4 and BatCoV-HKU5 contain N-terminal extensions of 50 and 51 amino acids, respectively, relative to that of MERS-CoV (17) (Fig. 3B). Similar to JMHV and MHV-A59, the MERS-CoV I protein is truncated at the N-terminus as a result of an in-frame stop codon upstream of the coding sequence of MERS-CoV I protein (17). Sequence conservation is again observed between the N-terminal domain of the I protein of BatCoV-HKU4 and BatCoV-HKU5 and the coding amino acid sequence upstream of the start codon of MERS-CoV I protein, albeit to a lesser extent compared to the I proteins of JHMV and MHV-A59. In addition to the N-terminus truncation, the MERS-CoV I protein is also truncated at the C-terminus relative to those of BatCoV-HKU4 and BatCoV-HKU5 (17). The effect of these truncations on I protein function and their role in virus evolution and host adaptation, if any, remain areas that require future investigation.

In contrast to embecoviruses and merbecoviruses*,* closely related bat and human sarbecoviruses share extensive similarity in their I protein sequences (65–67), suggesting that I protein changes may not be necessary for the initial zoonotic spillover of sarbecoviruses. However, mutations in the SARS-CoV-2 I protein coding sequence have been observed during the coronavirus disease 2019 pandemic. The I protein of the Gamma variant encodes a Q77E mutation while that of the Delta variant encodes a T60A mutation (24, 68, 69). P10S mutation in the I protein was identified in early Omicron variants with further deletions observed in some of the subsequent Omicron subvariants including BA.1, BA.2, BA.4, and BA.5 (68). The effect of these mutations in altering SARS-CoV-2 I protein structure and function, if any, remains unclear. The emergence of these mutations also suggests that the I protein has been evolving during the pandemic with the caveat that these mutations also altered the coding sequence of the N protein.

## CONCLUSION

The betacoronavirus I protein is a multifunctional protein that remains under-characterized. Our current knowledge of the I protein is predominantly centered around IFN antagonism. While this is an important function for the I protein, abundant evidence suggests that it has additional uncharacterized functions that remain undiscovered. The close interaction reported between the I proteins of several betacoronaviruses and TOM70, a mitochondria-associated protein, indicates that the I protein may affect mitochondrial function. The consequence of I protein-mediated alteration of mitochondrial function on pathogenesis merits further study. Furthermore, the I protein plays a virus-specific role in pathogenesis. Future studies on the disparate role of I protein-mediated pathogenesis in a virus-specific context would offer further insights underlining the factors contributing to either the protective or detrimental nature of the protein.
